# Sensitivity of Compressed Composite Channel Columns to Eccentric Loading

**DOI:** 10.3390/ma15196938

**Published:** 2022-10-06

**Authors:** Pawel Wysmulski, Hubert Debski, Katarzyna Falkowicz

**Affiliations:** Department of Machine Design and Mechatronics, Faculty of Mechanical Engineering, Lublin University of Technology, 36 Nadbystrzycka Street, 20-618 Lublin, Poland

**Keywords:** buckling, critical state, eccentricity of load, thin-walled structures, composites, finite element method

## Abstract

This study investigated short thin-walled channel columns made of carbon/epoxy laminate. Columns with two multi-ply composite layups [0/45/−45/90]_s_ and [90/−45/45/0]_s_ were tested, with each layup having eight plies symmetric to the midplane. The columns were subjected to compressive loads, including an eccentric compressive load applied relative to the center of gravity of their cross-section. Simple support boundary conditions were applied to the ends of the columns. The scope of the study included analyzing the effect of load eccentricity on the buckling mode, bifurcation load (idealized structure), and critical load (structure with initial imperfections). The critical load for the actual structure was determined with the use of approximation methods, based on experimental postbuckling equilibrium paths. In parallel with the experiments, a numerical analysis was conducted using the finite element method and Abaqus^®^ software (Dassault Systèmes, Vélizy-Villacoublay, France). The first stage of the numerical analysis consisted of solving an eigenproblem, in order to determine the mode of the loss of structural stability and to calculate the bifurcation loads for structures under axial and eccentric compression. The second stage of the numerical analysis involved examining the non-linear state of pre-deflected structures. Numerical postbuckling equilibrium paths were used to estimate the critical loads with an approximation method. The experimental results were used to validate the numerical models. This made it possible to determine the effect of compressive load eccentricity on the buckling mode and critical load of the tested structures. The results confirmed that compressive load eccentricity had a significant impact on the load-carrying capacity in the postbuckling range. This may potentially lead to premature damage to composite materials and, ultimately, to a reduced load-carrying capacity of structures.

## 1. Introduction

Bearing elements in modern thin-walled structures are predominantly made of advanced composite materials characterized by a low specific weight and high mechanical performance [1,2,3,4,5,6,7,8,9]. This primarily concerns thin-walled stiffeners, which are usually designed as open and closed profiles, with complex cross-sectional shapes. The main role of these structural members is to transfer axial and bending loads, which means that they are exposed to buckling, particularly under compressive loads. Although a loss of stability by these members may cause local weakening in a load-bearing structure itself, this poses no direct threat to the safe performance of such structures [10,11,12,13]. Studies have shown [13,14,15,16,17,18] that thin-walled composite structural members can continue carrying loads in the buckled state, provided that the buckling is elastic and that the postbuckling equilibrium path is stable [19,20,21,22,23,24,25,26]. Considering the above, in addition to investigating the buckling of structural members, it is also necessary to investigate the postbuckling behavior of entire structures and their load-carrying capacity in a buckled state. Stability loss in thin-walled stiffeners usually takes the form of local buckling of the walls and web of the thin-walled column, and this is manifested by the occurrence of a certain number of half-waves along the column axis [27,28,29,30,31,32,33,34]. The load that causes columns to buckle is known as the critical load, its value being dependent on many factors. These factors include the following: the column’s cross-sectional shape and dimensions, its length, boundary conditions, and—for composite materials—the ply layup in the column’s walls and web [35,36,37,38,39]. Knowledge of the critical load value is of vital importance when designing thin-walled structures, because it allows taking measures to prevent these structures from buckling within the service load range. The determination of critical loads for actual (not idealized) structures is complicated, which significantly impedes the design of such structures. The critical load is determined by approximation methods [39,40,41], which make it possible to determine an approximate value of the critical force value based on the postbuckling equilibrium path of a given structure; this path is usually plotted as the load versus displacement perpendicular to the column’s walls. The critical load also depends on the extent of geometrical imperfections, which usually result from the manufacturing inaccuracy of an actual structure. One other important aspect of the design of thin-walled structures concerns the real loads, which in most cases differ from the idealized theoretical loading conditions. One of the key problems in this respect is the load eccentricity resulting from, for example, assembly inaccuracies or unstable boundary conditions. The behavior of a structure under eccentric loading significantly differs from the idealized axial loading response. Load eccentricity may lead to a premature loss of stability, with buckling occurring within the service load range. Changes may also occur in the way the walls and web of a column are loaded, because the eccentricity of the force application induces additional flexural loads that may be a direct cause of premature buckling. If a thin-walled structure enters into the postbuckling state too early, this may result in a significant reduction in its strength parameters and thus lead to a faster failure of its load-carrying members. This is all the more dangerous for actual structure performance, because the vast majority of design solutions do not take the above fact into consideration at the design stage. Most structures are usually designed on the assumption that their structural members are under idealized loading conditions. This research undertook to determine the qualitative and quantitative effect of compressive load eccentricity on the buckling and postbuckling behavior of thin-walled composite columns. In the study, different load eccentricity values were tested using experimental and numerical methods. It is also worth mentioning that this study involved using the popular finite element method, which is widely used in many fields [42,43,44,45,46,47,48,49].

## 2. Object of the Study

**Figure 1:**
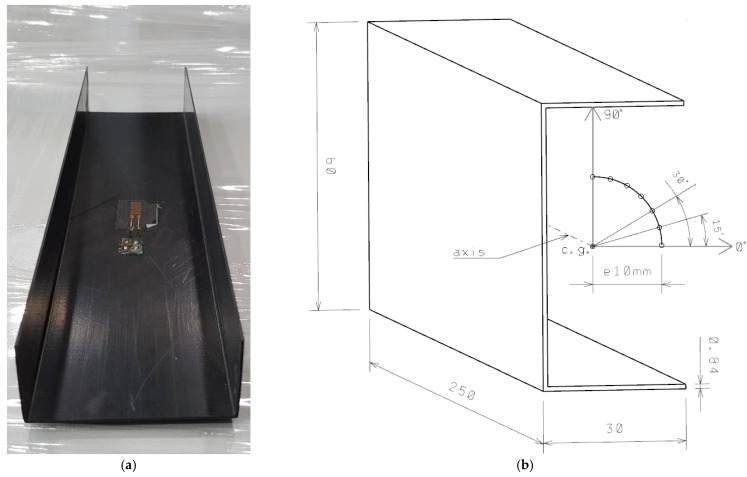
Channel column: (**a**) actual specimen, (**b**) geometrical model of a column under eccentric compression.

The object of this study were short thin-walled columns with a channel cross-section under axial and eccentric compression. The columns were standard thin-walled structures with perpendicular walls; they were flat plate elements joined by their longer ends [33,38,39,50,51,52,53,54]. The columns were fabricated from epoxy/carbon laminate by autoclaving (Figure 1a). The composite channel columns had the following layups: C1 [0/45/−45/90/90/−45/45/0]_T_ and C2 [90/−45/45/0/0/45/−45/90]_T._ The laminate consisted of eight plies arranged symmetrically relative to the midplane. The columns had the following overall dimensions: 60 × 30 × 250 × 0.84 mm—Figure 1b.

A schematic design of a channel column under an eccentric compressive load is shown in Figure 1b. Eccentricity was induced by moving the point of compressive force application relative to the longitudinal axis of the column. This point was moved from the center of gravity of the column’s cross-section to the 0° axis by a value of e = 10 mm, and then the column was gradually rotated by 0° < e = 10 mm < 90° every 15° relative to its axis (the point of compressive force application was changed in an arc with a radius of R = 10 mm, with its midpoint located in the center of gravity of the column—Figure 1b). The mechanical properties of a single composite ply, which were determined experimentally in compliance with the relevant ISO standard, were as follows: Young’s modulus along the fiber direction—*E*_1_ = 143.5 GPa, Young’s modulus perpendicular to the fiber direction—*E*_2_ = 5.83 GPa, Poisson’s ratio on the ply plane—*ν*_12_ = 0.36, and Kirchhoff’s modulus—*G*_12_ = 3.85 GPa.

## 3. Methodology of the Study

In this study, experimental and numerical methods were employed to investigate the buckling and early postbuckling responses of thin-walled composite structures under axial and eccentric compression. Experiments were performed up to approx. 200% of the numerical bifurcation load value. The objective of the study was to investigate the buckling behavior of these columns and to determine their postbuckling characteristics, describing the relationship between compressive load and deflection perpendicular to the column wall in the early postbuckling regime. This made it possible to assess the effect of compressive load eccentricity on the stability of the channel section columns. This paper is an extension of the team’s research, which was presented in a previous work [55].

### 3.1. Experimental

**Figure 2:**
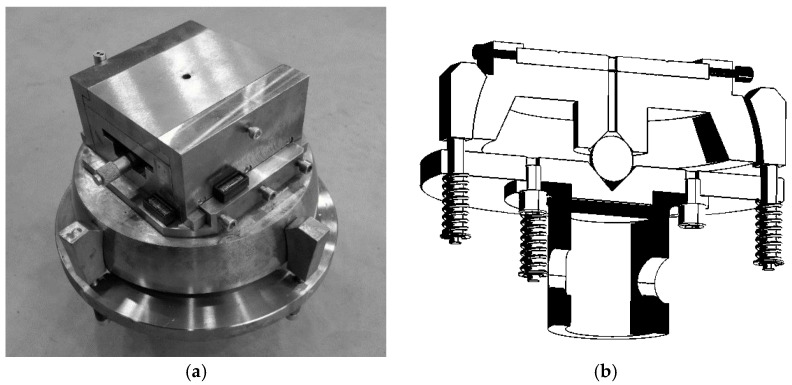
Experimental setup: (**a**) jointed head, with attached table for precision eccentricity of load, (**b**) cross-section model of the ball-and-socket joint head.

Experiments were conducted at ambient temperature on a static testing machine, Zwick-100 (Zwick Roell, Ulm, Germany), with the upper cross-bar travel speed maintained constant at 2 mm/min. During the experimental tests, the deformations of the column web were recorded using resistance strain gauges, taped on both sides of the web at the location of the expected largest deflections. In addition, the deflections of the column walls in the direction perpendicular to the wall plane were measured using a laser sensor. These measurements made it possible to determine the postbuckling characteristics of the columns and thus describe their postbuckling behavior for the tested loading conditions. The column was simply-supported at both ends, with ball-and-socket joint heads mounted on the pins of the testing machine—Figure 2b.

**Figure 3:**
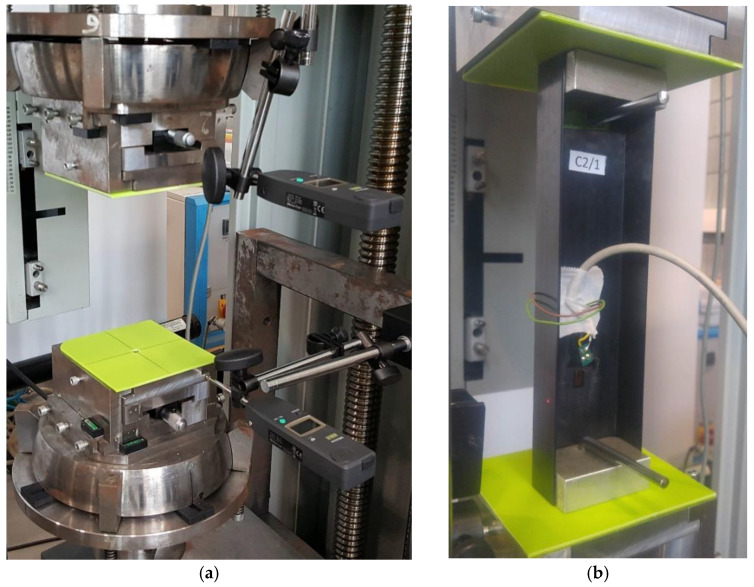
Experimental setup: (**a**) calibration of ball-and-socket joint head position, (**b**) position of a specimen on the sliding tables.

Specially designed sliding tables were mounted on the heads, in order to ensure that the eccentricity values were entered accurately via positioning the column relative to the static axis of the testing machine (Figure 2a). The centering of the channel column with respect to the pins of the testing machine was done by using plates with holes drilled at the center of gravity of the cross-section (Figure 3b). In order to ensure control of the load eccentricity realization with an accuracy of to 0.01 mm, an additional measuring system using electronic sensors was used (Figure 3a). The experiments were conducted for one axial compressive load case and two cases of eccentric loading: e10mm_0° and e10mm_90°. As a result, it was possible to validate the numerical FEM models for the extreme eccentricity values. Critical loads for the actual structure were calculated based on postbuckling equilibrium paths using approximation methods. Figure 3b shows the physical model of a channel column mounted in the heads of the testing machine, as well as the plates for positioning the column’s center of gravity relative to the axis of the testing machine.

During the tests, the course of the compression force, the deformation of the column web, and the deflection of the column walls in the perpendicular direction were registered using a laser sensor. The resulting equilibrium post-critical paths, which determine the dependence of the compressive load on the deflection of the P-w column walls, made it possible to determine the value of the critical load and to assess the performance of the structure in the in the post-critical range.

### 3.2. Numerical Analysis

In parallel with the experiments, numerical simulations were performed, to develop FEM models for analyzing the effect of the axial compressive load and eccentric load applied in a range of 0° < e = 10 mm < 90° every 15° on the tested structures. The columns were discretized, with the use of shell elements, and each element having three translational and three rotational degrees of freedom in every node. The numerical models of the columns were constructed with S8R finite elements, which are eight-node elements with a quadratic shape function and reduced integration [30,33,38,39]. The plates reproducing the simple support on the column ends were modelled using four-node rigid finite elements, R3D4.

**Figure 4:**
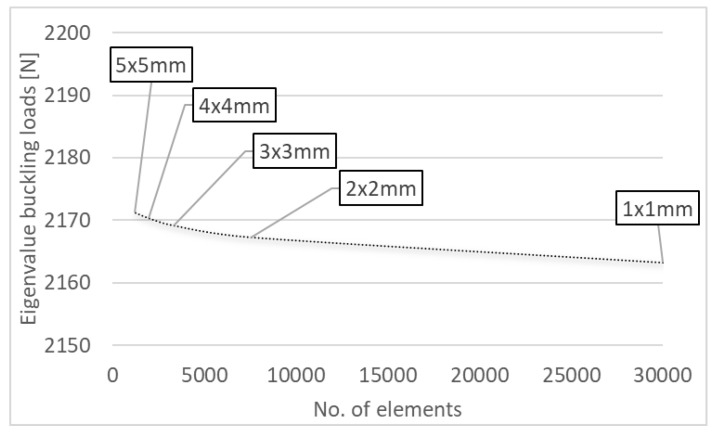
The convergence test results.

To investigate the effect of meshing size, this study tested five different element sizes: from 1 × 1 mm to 5 × 5 mm, for the chosen channel column. The mesh convergence was established by increasing the mesh density in each part of the model. It was observed that there were no considerable changes in load response between a 1-mm and 5-mm element size (Figure 4), but the processing time was considerable and an increment in mesh density was unnecessary, as shown in Figure 4.

The designed numerical models had a uniform finite element mesh, with each finite element having a size of 4 × 4 mm. In effect, the individual walls of the column could be divided in a uniform manner by generating a constant-density mesh. This approach made it possible to examine the strains and stresses occurring in the compressed thin-walled structures.

**Figure 5:**
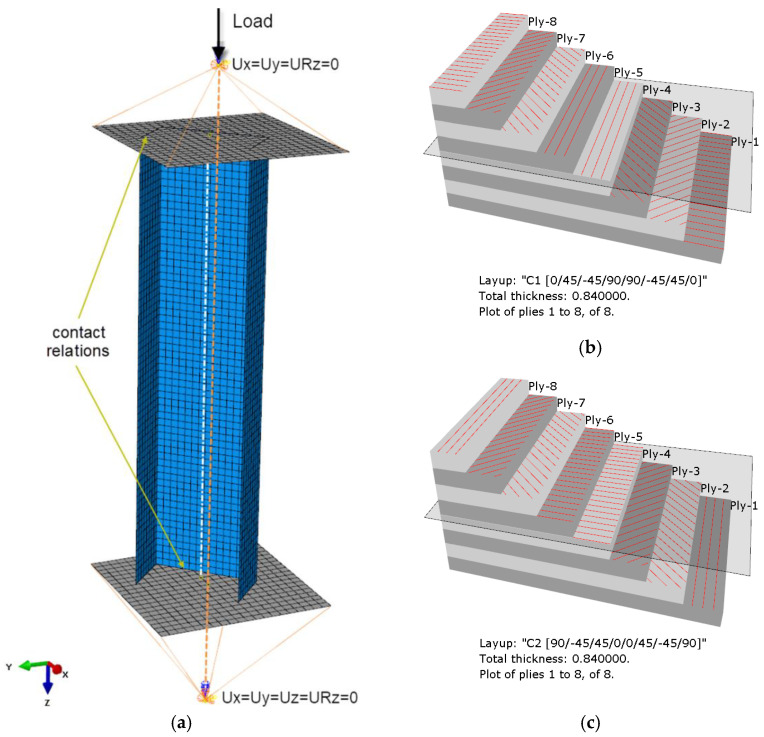
FEM analysis: (**a**) discrete model of a column and the boundary conditions for the eccentricity value of e10mm_45, (**b**) layup C1, (**c**) layup C2.

The FEM model attempted to represent the actual support of the column at both ends of the testing machine (Figure 5a). Reference points corresponding to the position of the centers of gravity of the ball joints of the mounting heads were used, where simple support boundary conditions were defined. Two translational degrees of freedom (*Ux* = *Uy* = 0) and rotation relative to the axis of the column (*URz* = 0) were constrained at the point reproducing the upper ball-and-socket joint, whereas three translational degrees of freedom (*Ux* = *Uy* = *Uz* = 0) and rotation (*URz* = 0) were constrained at the reference point for the lower ball-and-socket joint. The reference points were linked with the rigid plates supporting the columns ends, by coupling all degrees of freedom of the points and the plates. The column was loaded with a compressive force concentrated at the upper reference point. A given value of the eccentricity parameter “e” was applied by changing the position of the column model relative to the rigid plates and displacing it perpendicular to the web (Figure 1b) by a value of 10 mm, and then rotating it with a fixed value every 15°. Contact relations were defined between the column ends and the plates. The structure of the laminate structure was modelled using the layup–ply technique, which made it possible to reproduce the layup of the composite material and ensure a uniform thickness of the finite elements by defining the ply thickness, material type, and fiber orientation (C1—Figure 5b, C2—Figure 5c).

### 3.3. Analysis of the Early Postbuckling Range

FEM numerical calculations were performed in two stages. The first involved solving an eigenproblem, to determine both the bifurcation load of an idealized structure using the minimum potential energy principle [56] and the corresponding buckling mode—Figure 4. For such a system, an eigenvalue problem was solved using the following equation [57]:

(1) $$\left(\left[K\right]+\lambda_{i}\left[H\right]\right){\left\{\psi \right\}}_{i}=0$$

where [*K*] is the structural stiffness matrix, [*H*] is the stress stiffness matrix, *λ_i_* is the *i*-th eigenvalue, and *ψ* is the *i*-th eigenvector of displacement.

Equation (1) is satisfied if the eigenvector of displacement is equal to zero, or if the determinant of the term in brackets is equal to zero. When {*ψ*}*_i_* = 0, this is a trivial solution, and it is out of interest. This means that the structure remains in the initial state of equilibrium. The term in brackets in Equation (1) gives the following solution:

(2) $$\left|\left[K\right]+\lambda_{i}\left[H\right]\right|=0$$

Equation (2) represents the eigenvalue problem that allows finding the *n* values of the buckling load multiplier *λ* and the corresponding buckling mode shape.

**Figure 6:**
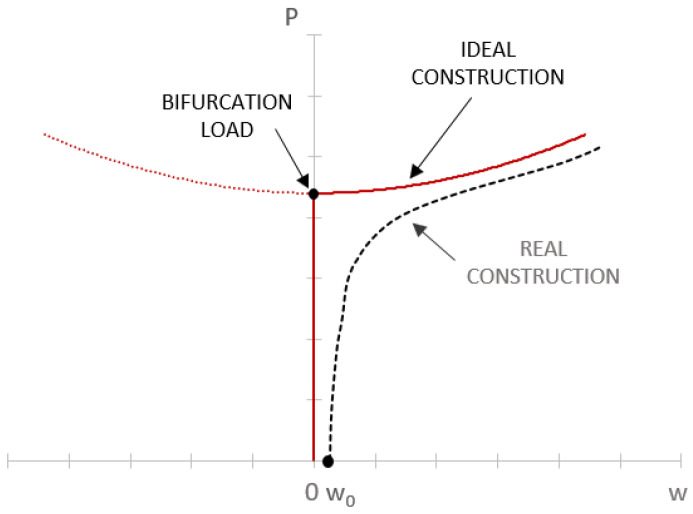
Postbuckling equilibrium paths of two structures: idealized, and actual (with initial geometric imperfections described by an amplitude *w*_0_).

The other stage of the numerical calculations was a nonlinear analysis of the early postbuckling response up to 200% of the bifurcation load. A nonlinear analysis was performed, with the use of a model of the structure with initial geometric imperfections reflecting the lowest buckling mode; the initial amplitude of these imperfections being *w*_0_ = 0.1 mm—Figure 6. The value of the initial imperfections was determined by experimental validation of the numerical postbuckling characteristics of the tested structures. The nonlinear stability problem was solved using the Newton–Raphson method. The other stage of the calculations involved determining the postbuckling equilibrium paths, which were used as a basis for determining the critical loads of the structures with initial geometric imperfections.

### 3.4. Method of Determining the Critical Load of a Structure with Initial Imperfections

Various types of inaccuracies could be observed in the experiments, resulting from different factors, such as geometrical imperfections, the load application, and boundary conditions. In effect, it was difficult to determine the exact critical load values for the thin-walled structures. In cases such as these, approximation methods must be employed to determine critical loads based on the obtained experimental and numerical postbuckling equilibrium paths. In this study, critical loads were determined using an approximation method developed by Koiter.

The Koiter method was used to approximate the experimental and numerical postbuckling equilibrium paths describing the relationship between the load and deflection perpendicular to the column wall. The postbuckling equilibrium paths *P-w* for the early postbuckled state were approximated using a quadratic function, having the form [40]:

(3) $$P=P_{cr}\frac{a_{2}}{a_{0}}w^{2}+P_{cr}\frac{a_{1}}{a_{0}}+P_{cr}$$

where *a*_0_, *a*_1_, and *a*_2_ are the parameters of the function, *P* is the load, *P_cr_* is the value of an unknown critical load, and *w* is the increase in deflection measured perpendicular to the column’s wall.

**Figure 7:**
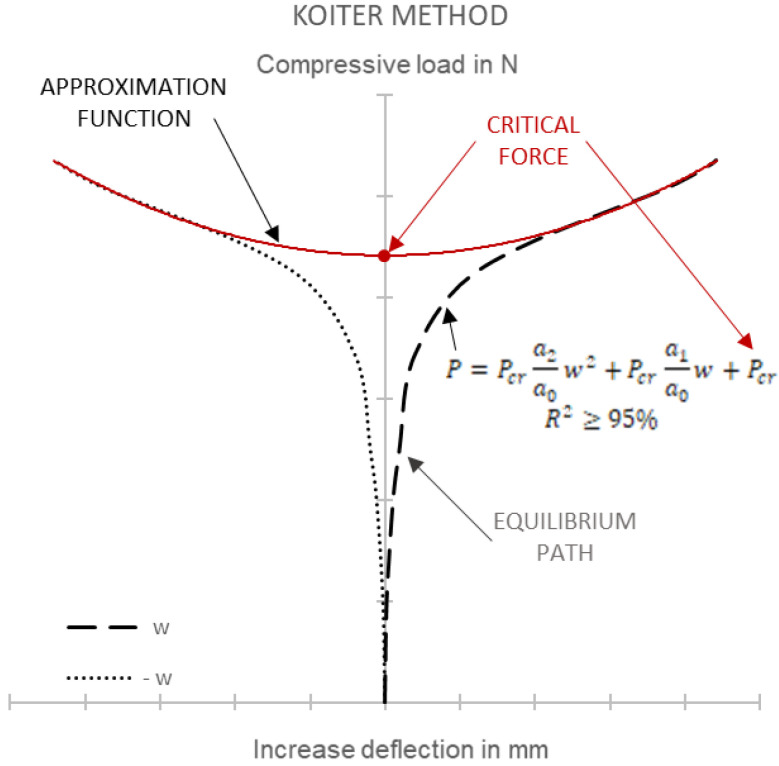
Equilibrium path approximation using Koiter’s method.

According to Koiter’s method, the critical force is defined as a point of intersection between the quadratic function and the vertical axis of the coordinate system in the *P-w* plot (Figure 7). The accuracy of the critical load value depends on the employed approximation range, which means that for a stable postbuckling equilibrium path, the leading coefficient of a quadratic polynomial must be positive. Approximation accuracy is estimated with a correlation coefficient R^2^. The correlation coefficient value shows whether the approximation function agrees with a selected range of the experimental approximated curve. The higher the correlation coefficient value, the more accurate the approximation process becomes. With the approximation method employed in this study, the minimal value of the correlation coefficient was equal to R^2^ ≥ 95%.

## 4. Results and Discussion

**Figure 8:**
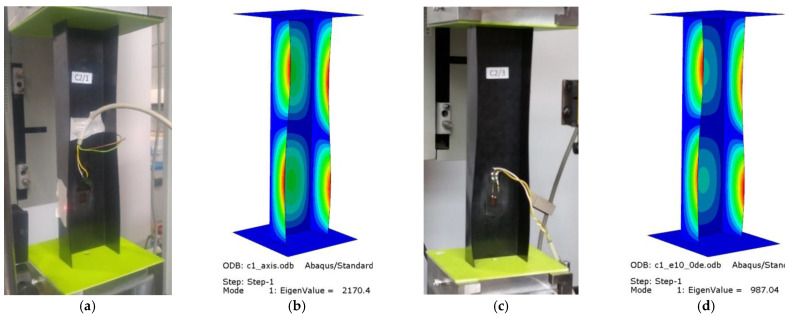

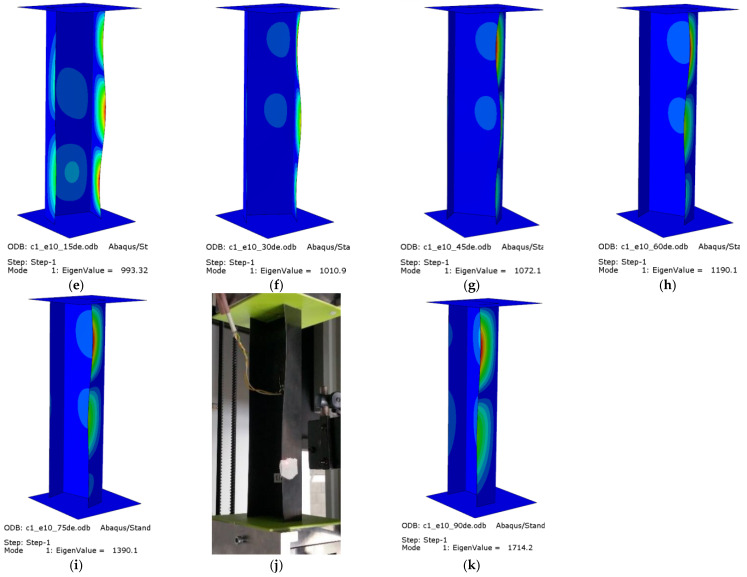
Buckling modes for C1 [0/45/−45/90]_s_: (**a**) axis EXP, (**b**) axis FEM, (**c**) e10_0° EXP, (**d**) e10_0° FEM, (**e**) e10_15° FEM, (**f**) e10_30° FEM, (**g**) e10_45° FEM, (**h**) e10_60° FEM, (**i**) e10_75° FEM, (**j**) e10_90° EXP, (**k**) e10_90° FEM.

**Figure 9:**
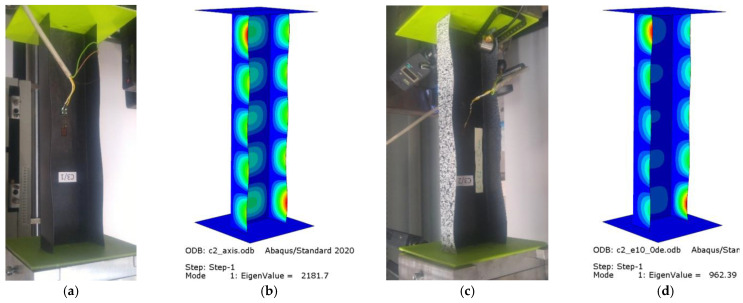

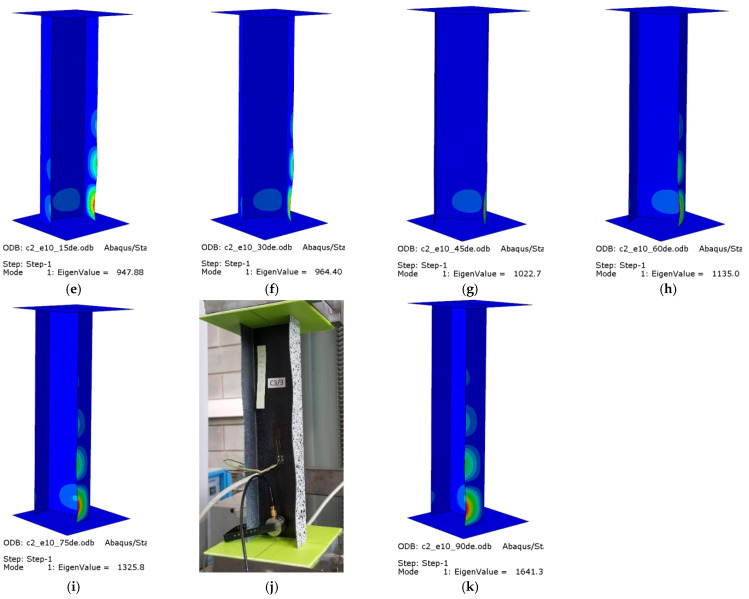
Buckling modes for C2 [90/−45/45/0]_s_: (**a**) axis EXP, (**b**) axis FEM, (**c**) e10_0° EXP, (**d**) e10_0° FEM, (**e**) e10_15° FEM, (**f**) e10_30° FEM, (**g**) e10_45° FEM, (**h**) e10_60° FEM, (**i**) e10_75° FEM, (**j**) e10_90° EXP, (**k**) e10_90° FEM.

The experiments and numerical analysis of thin-walled composite channel columns under axial and eccentric compression provided data about the effect of compressive load eccentricity on the buckling and postbuckling behavior of the tested columns. The results allowed for an examination of the buckling state based on the buckling modes of the actual and numerical structures. Figure 8 shows the experimental and numerical buckling modes obtained for C1 [0/45/−45/90/90/−45/45/0]_T_, while Figure 9 shows the experimental and numerical buckling modes for C2 [90/−45/45/0/0/45/−45/90]_T_.

For the axially compressed column C1 (Figure 8a,b), local buckling of the web and walls occurs, having the form of two longitudinal half-waves symmetric to the longitudinal symmetry plane of the column. There is no change in the buckling mode of the C1 column when the eccentricity value of e10mm_0° is applied; however, one can observe increased loading of the column’s walls. This results from the fact that the point of compressive force application was moved, which led to unloading of the web of the column (Figure 8c,d). The eccentricity value of 15° < e = 10 mm < 75° causes a total change in the buckling mode, from two to three half-waves (Figure 8e–h). For the extreme C1 eccentricity case of e10mm_90°, local buckling returns to the initial mode, i.e., two half-waves.

The axial compression of C2 (Figure 9a,b) is characterized by local buckling of the web and walls, with the buckling having the form of five half-waves symmetric to the symmetry plane of the column. For the eccentricity applied from the web of the C2 column, i.e., e10mm_0° (Figure 9c,d), the number of half-waves on the column walls and web remains the same, but—as in the case of C1—unloading of the web and increased loading of the walls occurs at the same time. For the eccentricity value of 15° < e = 10 mm < 90° there is a change in the buckling mode; the half-waves on the unloaded wall and the web disappear, while the half-waves in proximity of the bottom end of the column become more visible (Figure 9e–k). The experimental and numerical buckling modes showed a qualitative agreement.

To determine the quantitative relationship between load eccentricity and buckling, the obtained bifurcation loads describing the lowest buckling modes of the compressed composite columns were analyzed numerically. Table 1 shows the FEM eigenvalue buckling loads obtained for these structures. The difference between the eigenvalue buckling loads of C1 and C2 for the axial compression case was insignificant and only amounts to 0.5%, whereas the greatest difference of 4.6% can be observed for the eccentricity value of e10mm_30°. That means that both structures, which differ with respect to the position of the 0° and 90° plies, have a similar stiffness and thus undergo buckling under similar critical loads.

**Figure 10:**
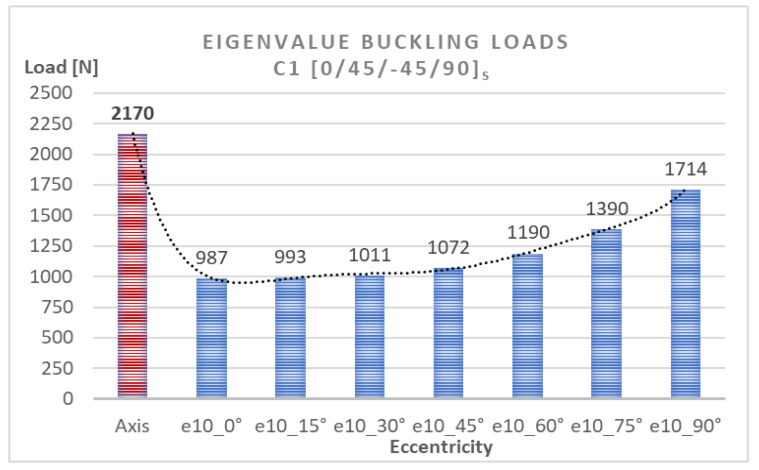
Eigenvalue buckling loads for C1 [0/45/−45/90]_s_.

**Figure 11:**
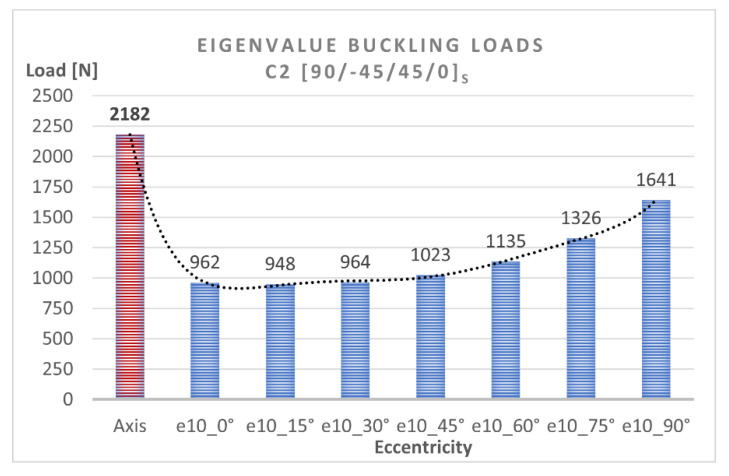
Eigenvalue buckling loads for C2 [90/−45/45/0]_s_.

The numerical bifurcation loads obtained for the tested eccentric compression loads are shown graphically in Figure 10 and Figure 11. The C1 column exhibits a clear decrease in the bifurcation load under eccentric loading—Figure 10. The highest drop in the bifurcation load can be observed for C1 under e10mm_0° and amounts to 55% compared to the axial compression case, while the minimum difference is the 21% observed for C1 under e10mm_90°. The C2 column [90/−45/45/0]_s_ is slightly more sensitive to load eccentricity, ranging <33% ÷ 57% >—Figure 11. The highest decrease in the bifurcation load of C2 can be observed for the eccentricity value of e10mm_15°. The results demonstrate that C1 [0/45/−45/90]_s_ and C2 [90/−45/45/0]_s_ had similar bifurcation loads under the applied eccentric load conditions.

**Figure 12:**
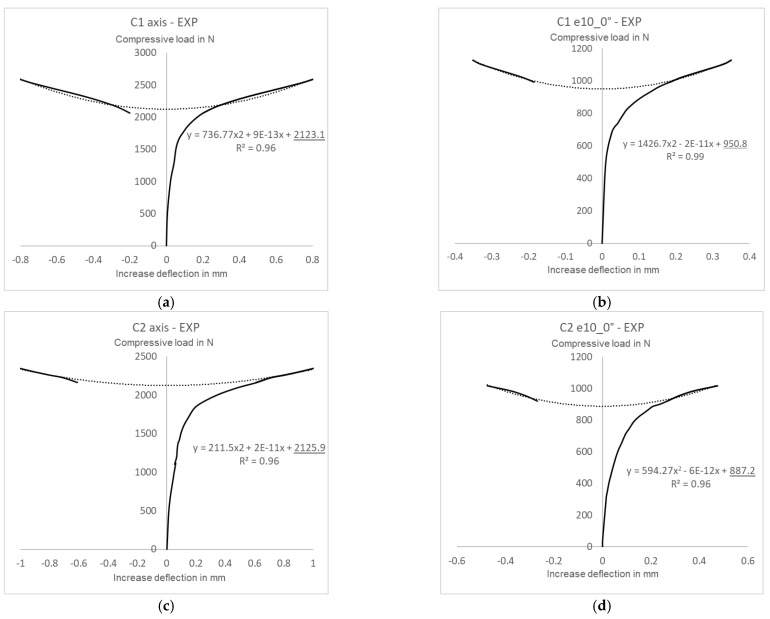
Experimental critical loads for structures with initial imperfections: (**a**) C1 axis, (**b**) C1 e10_0°, (**c**) C2 axis, (**d**) C2 e10_0°.

**Figure 13:**
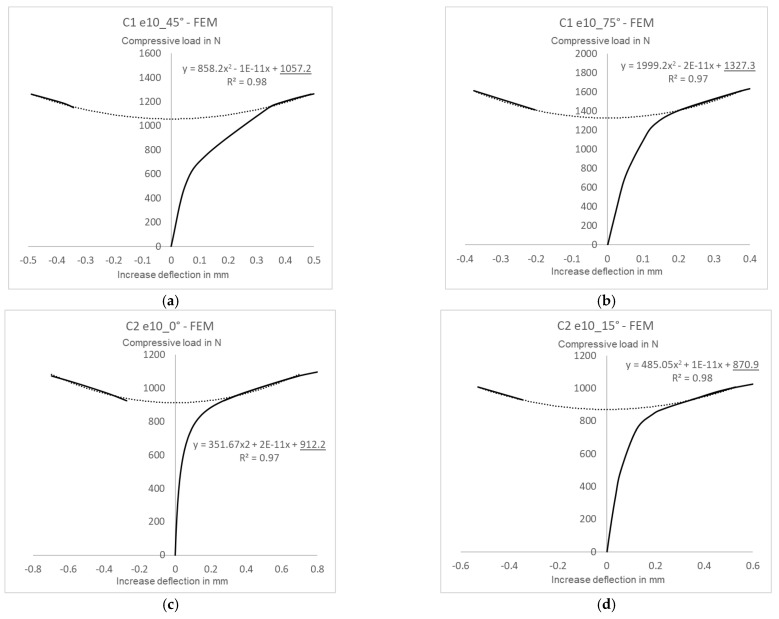
Numerical critical loads for structures with initial imperfections: (**a**) C1 e10_45°, (**b**) C1 e10_75°, (**c**) C2 e10_0°, (**d**) C2 e10_15°.

The critical loads of the pre-deflected structures were calculated based on the experimental and numerical postbuckling equilibrium paths describing a load-deflection relationship measured perpendicularly to the channel column wall. A selection of the experimental approximated characteristics are shown in Figure 12, while the numerical ones are shown in Figure 13.

The postbuckling equilibrium paths describing the relationship between a compressive load *P* and a deflection *w* served as the basis for determining critical loads using Koiter’s approximation method [38,39,40]. For all tested cases the approximated experimental curve range included some portion of the early postbuckling path, from a clear inflection point of the load-deflection line, until the end of the experimental curve. For all analyzed cases, the coefficient of correlation was maintained at a very high level of R^2^ ˃ 95%. The numerical and experimental critical loads obtained with this method are given in Table 2.

The approximated values of the experimental and numerical critical loads for the structures with initial imperfections show a good agreement. The greatest difference between the critical load values of C1 [0/45/−45/90]_s_ can be observed for the eccentricity of e10mm_90°, with this difference amounting to about 1%, whereas for C2 [90/−45/45/0]_s_ the difference is slightly higher, yet does not exceed 3% (C2 e10mm_0°). It should be emphasized that the experimental critical loads are the lower estimation of the FEM critical load values, which results from the structural idealization of the numerical model.

**Figure 14:**
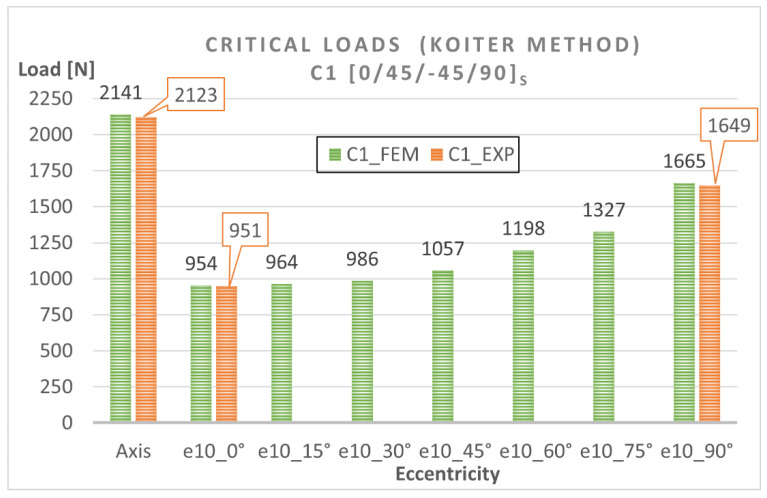
Eccentricity versus critical load for C1 [0/45/−45/90]_s_.

**Figure 15:**
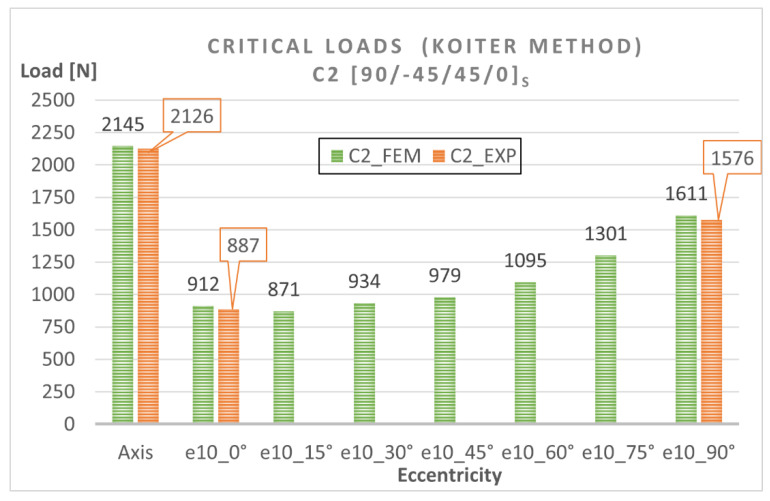
Eccentricity versus critical load for C2 [90/−45/45/0]_s_.

To estimate the effect of load eccentricity on the critical load of the analyzed columns (C1 and C2), diagrams illustrating the relationship between the critical load and eccentricity were created (Figure 14 and Figure 15). The diagrams show a very good agreement between the numerical results and the experimental findings, which confirms that the numerical models were correct.

**Figure 16:**
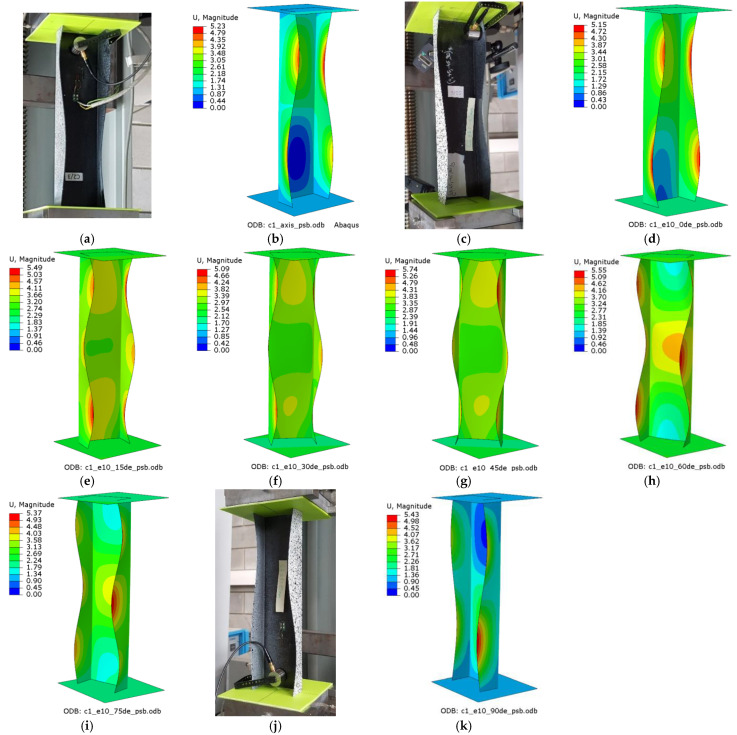
Early postbuckling modes for C1 [0/45/−45/90]_s_: (**a**) axis EXP, (**b**) axis FEM, (**c**) e10_0° EXP, (**d**) e10_0° FEM, (**e**) e10_15° FEM, (**f**) e10_30° FEM, (**g**) e10_45° FEM, (**h**) e10_60° FEM, (**i**) e10_75° FEM, (**j**) e10_90° EXP, (**k**) e10_90° FEM.

**Figure 17:**
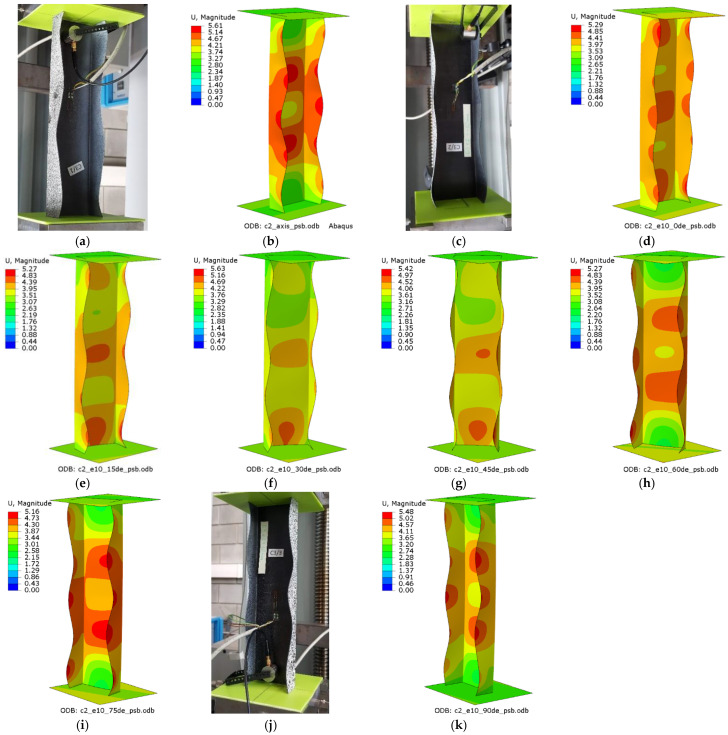
Early postbuckling modes for C2 [90/−45/45/0]_s_: (**a**) axis EXP, (**b**) axis FEM, (**c**) e10_0° EXP, (**d**) e10_0° FEM, (**e**) e10_15° FEM, (**f**) e10_30° FEM, (**g**) e10_45° FEM, (**h**) e10_60° FEM, (**i**) e10_75° FEM, (**j**) e10_90° EXP, (**k**) e10_90° FEM.

The results of the postbuckling behavior of the compressed channel columns demonstrate that the deflection reflecting the lowest buckling modes increases when increasing the compressive load. The tests were conducted up to 200% of the bifurcation load. Figure 16 and Figure 17 show the buckling modes for the two compressed channel columns: C1 [0/45/−45/90]_s_ and C2 [90/−45/45/0]_s_.

**Figure 18:**
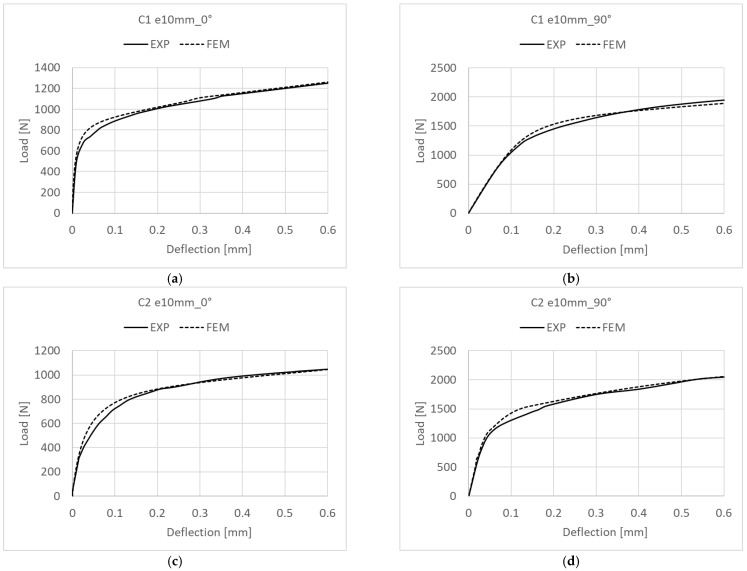
Experimental and numerical postbuckling equilibrium paths for: (**a**) C1 e10mm_0°, (**b**) C1 e10mm_90°, (**c**) C2 e10mm_0°, (**d**) C2 e10mm_90°.

The postbuckling behavior of the tested columns was analyzed by comparing the experimental and numerical postbuckling equilibrium paths obtained for these structures. The experimental equilibrium paths describing the relationship between the load and deflection were created based on the load results and measurements made with a laser beam pointed perpendicularly at a half-wave on the channel wall with the largest deflection. The numerical equilibrium paths were generated by measuring the column wall’s deflection perpendicularly, with the measurements made at the same point as in the experiments. Figure 18 compares the experimental and numerical paths obtained for C1 and C2.

**Figure 19:**
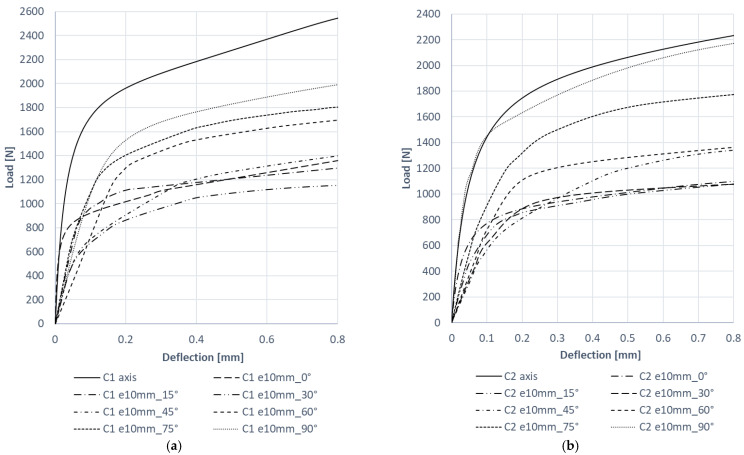
Load eccentricity versus postbuckling behavior for: (**a**) C1, (**b**) C2.

The experimental and numerical results show a good agreement. Similarly to buckling, the experimental postbuckling equilibrium paths show a slight reduction in the column’s stiffness compared to the numerical paths. Nevertheless, the experimental and numerical paths indicate a stable behavior of the structures, which confirms that the structures can carry loads in the postbuckling range. Figure 19 shows the numerical postbuckling equilibrium paths obtained for C1 and C2, and considering the eccentricity.

The results demonstrate that the postbuckling stiffness of C1 and C2 decreased when eccentricity was applied. Regarding C1 (Figure 19a), the maximum decrease in the stiffness of this structure under eccentric load was observed for e10mm_30° and amounted to 57% (compared to the axial compression case). As for C2 (Figure 19b), the eccentricity of e10mm_0° caused the highest decrease in the column’s stiffness, of 52%. An analysis of the other cases tested demonstrated a 10% increase in the stiffness of C2 for the eccentricity value of e10mm_90°. The smallest decrease in the stiffness of eccentrically loaded C1 and C2 was observed for the eccentricity value of e10mm_90°.

The high costs of producing carbon–epoxy composite profiles make tests of this type expensive. This is a major limitation for scientists who research this type of structure. It should be added that FEM modelling may be interesting for considering the progressive failure and failure processes in composites [58,59,60,61,62,63,64,65]. For this reason, future research directions will focus on the analysis of the post-critical state in the full range of loads leading to the damage and failure of the composite structure.

## 5. Conclusions

This study investigated the effect of compressive load eccentricity on the stability and postbuckling behavior of thin-walled composite channel columns. A combined qualitative and quantitative analysis of the effect of the compressive load eccentricity on critical loads and postbuckling equilibrium paths of these structures was performed. The results demonstrated that the eccentricity applied perpendicular and parallel to the web of the column did not change its buckling mode, when compared to the axial compression case. The buckling mode would only change if the eccentricity was applied simultaneously in two directions (in an angle range of 15° ÷ 75°). The weakening of the column cross-section and the change in the form of buckling was due to an increase in the additional bending state, resulting from the eccentricity of the load.

The results showed that the tested ply layups had an insignificant effect on the buckling behavior of the columns. Fiber orientation had a negligible effect on the critical load. Despite changing the fiber orientations of the 0° and 90° plies, no significant differences were observed between the critical loads obtained for C1 and C2, irrespective of the eccentricity value. Nevertheless, a clear relationship was observed between the eccentricity and critical load. Compared to the axial compression case, the C1 column showed a significant decrease of 55% in the bifurcation load for the eccentricity value of e10mm_0°. The C2 column was even more sensitive to load eccentricity; the bifurcation load decrease was as high as 57% for the eccentricity value of e10mm_15°. Therefore, for the tested cases, load eccentricity may have caused premature buckling of the structures and thus considerably reduced their postbuckling load-carrying capacity. It should also be highlighted that the approach adopted in this study for determining the critical load based on postbuckling equlibrium paths was correct, as proven by the very high agreement between the experimental and numerical critical loads for two extreme eccentricity cases (axial and eccentric compression, e10mm_0° and e10mm_90°). This fact also confirms that the numerical models of the tested structures were correct.

The results also showed the good agreement of the experimental and numerical postbuckling equilibrium paths describing the relationship between the load and deflection measured perpendicular to the plane of the column wall. The results demonstrated that load eccentricity had a significant impact on structural stiffness in the postbuckling range. Compared to the axial compression case, the application of the eccentricity value of e10mm_30° caused a 57% reduction in the stiffness of C1, whereas the eccentricity of e10mm_0° resulted in a 52% decrease in the stiffness of C2. These results confirmed that the compressive load eccentricity had a significant impact on load-carrying capacity in the postbuckling range. This may potentially lead to premature damage of composite materials and, ultimately, to a reduced load-carrying capacity of structures. The results are of practical significance, particularly for the design of thin-walled composite structures that are exposed to unexpected inaccuracies, which may considerably affect their performance.

## Figures and Tables

**Table 1:** Eigenvalue buckling loads of the tested structures.

	Eigenvalue Buckling Loads [N]							
		Eccentricity						
	Axis	e10_0°	e10_15°	e10_30°	e10_45°	e10_60°	e10_75°	e10_90°
C1 [0/45/−45/90]s	2170	987	993	1011	1072	1190	1390	1714
C2 [90/−45/45/0]s	2182	962	948	964	1023	1135	1326	1641
Diference	−0.55%	2.53%	4.53%	4.65%	4.57%	4.62%	4.60%	4.26%

**Table 2:** Critical loads for structures with initial imperfections.

	Critical Loads (Koiter Method) [N]							
		Eccentricity						
	Axis	e10_0°	e10_15°	e10_30°	e10_45°	e10_60°	e10_75°	e10_90°
C1_FEM	2141	954	964	986	1057	1198	1327	1665
C1_EXP	2123	951	-	-	-	-	-	1649
Diference	0.84%	0.31%	-	-	-	-	-	0.96%
C2_FEM	2145	912	871	934	979	1095	1301	1611
C2_EXP	2126	887	-	-	-	-	-	1576
Diference	0.89%	2.74%	-	-	-	-	-	2.17%

## Data Availability

Not applicable.
